# TOAST stroke subtype classification in clinical practice: implications for the Get With The Guidelines-Stroke nationwide registry

**DOI:** 10.3389/fneur.2024.1375547

**Published:** 2024-03-22

**Authors:** Cecelia M. Rathburn, Katherine T. Mun, Latisha K. Sharma, Jeffrey L. Saver

**Affiliations:** Department of Neurology and Comprehensive Stroke Center, David Geffen School of Medicine at UCLA, Los Angeles, CA, United States

**Keywords:** ischemic stroke, TOAST, GWTG, registry, subtype classification

## Abstract

**Introduction:**

The TOAST (Trial of ORG 10172 in Acute Stroke Treatment) is the most commonly used ischemic stroke subtype classification system worldwide and a required field in the US National Get With The Guidelines-Stroke (GWTG-Stroke) registry. However, stroke diagnostics have advanced substantially since the TOAST classification was designed 30 years ago, potentially making it difficult to apply reliably.

**Methods:**

In this prospective diagnostic accuracy study, we analyzed consecutive ischemic stroke patients admitted to a Comprehensive Stroke Center between July–October 2021. Clinical practice TOAST classification diagnoses rendered by the stroke team in the electronic medical record (EMR) at discharge were retrieved from GWTG-Stroke registry and compared to a reference (“gold”) standard diagnosis derived from agreement between two expert raters after review of the EMR and patient imaging.

**Results:**

Among 49 patients; age was 72.3 years (±12.1), 53% female, and presenting NIHSS median 3 (IQR 1–11). Work-up included: brain imaging in 100%; cardiac rhythm assessment in 100%; cervical/cerebral vessel imaging in 98%; TTE ± TEE in 92%; and TCD emboli evaluation in 51%. Reference standard diagnoses were: LAA-6%, SVD-14%, CE-39%, OTH-10%, UND-M (more than one cause)-20%, and UND-C (cryptogenic)-10%. GWTG-Stroke TOAST diagnoses agreed with reference standard diagnoses in 30/49 (61%). Among the 6 subtype diagnoses, specificity was generally high (84.8%–97.7%), but sensitivity suboptimal for LAA (33%), OTH (60%), UND-M (10%), and UND-C (20%). Positive predictive value was suboptimal for 5 of the 6 subtypes: LAA (13%), SVD (58%), OTH (75%), UND-M (50%), and UND-C (50%).

**Discussion:**

Clinical practice TOAST classification subtype diagnoses entered into the GWTG-Stroke registry were accurate in only 61% of patients, a performance rate that, if similarly present at other centers, would hamper the ability of the national registry to provide dependable insights into subtype-related care. Development of an updated ischemic stroke subtype classification system, with algorithmic logic embedded in electronic medical records, is desirable.

## Introduction

Diagnosing etiologic subtypes of ischemic stroke is important for individual patient management, as a guide to subtype-specific therapeutics, to population health, and to delineate different and evolving patterns of disease in large populations. The most commonly used ischemic stroke subtype classification system worldwide is the Trial of ORG 10172 in Acute Stroke Treatment (TOAST) classification system (1).

One large-scale application of the TOAST classification system is the US nationwide Get with the Guidelines-Stroke (GWTG-Stroke) registry. Since March 2016, entering each patient’s TOAST classification at time of discharge has been a required data field. Discharge etiologic diagnoses documented in the electronic medical record (EMR) are abstracted and entered in the registry. This data has been used to determine the frequency of different ischemic stroke subtypes in the United States (US) (2).

However, stroke diagnostics have advanced substantially since the TOAST classification was designed 30 years ago, potentially making it difficult to now apply reliably, due to physician unfamiliarity with so old an instrument, improved understanding of stroke pathophysiology, and the advent of new imaging modalities. This study’s objective was undertaken to delineate the accuracy of contemporary TOAST classifications entered into the EMR and GWTG-Stroke by clinicians in routine practice compared with an expert reference standard.

## Methods

The study population for this prospective diagnostic accuracy study consisted of consecutive acute ischemic stroke patients admitted to the Ronald Reagan UCLA Medical Center (RRMC) between July 2021 and October 2021. RRMC is a Joint Commission—certified Comprehensive Stroke Center recognized by the American Heart Association for achieving GWTG-Stroke performance measures at the attainable highest rates (GWTG-Stroke Gold Plus and Target: Stroke Honor Roll Elite Awards). Final diagnosis of ischemic stroke diagnosis was confirmed through review of patient medical record. For each patient, the diagnoses of ischemic stroke subtype rendered clinically at the time of discharge and entered in the EMR and the GWTG—Stroke registry were compared with a reference standard ischemic stroke subtype diagnosis derived from two expert raters. Both approaches used the TOAST classification system. TOAST classification has five stroke subtype categories: (1) large vessel atherothromboembolic (LAA), (2) cardioembolic (CE), (3) small vessel disease (SVD), (4) stroke of other determined etiology (OTH), and (5) stroke of undetermined etiology (UND). For patient-level analyses, the stroke of undetermined etiology category was further subdivided into three subgroups: (a) Undetermined—more than one cause (UND-M); (b) Undetermined despite complete work-up (UND-C); and (c) Undetermined with incomplete work-up (UND-I). For etiology-level analyses, the UND-M category was further subdivided to identify each of the multiple etiologies individually.

Ischemic stroke subtype diagnoses were assigned in the medical record by the clinical stroke neurology team, with initial categorization by a neurology resident (PGY-2 or PGY-3) followed by final approval by the Stroke Neurology Faculty Attending (6 certified vascular neurologists). The Stroke Attendings all had at least once in their careers received clinical trial investigator training in TOAST diagnosis but did not undergo annual detailed retraining and certification in use of TOAST. Both resident and faculty physicians were instructed to employ TOAST classification to assign ischemic stroke etiology for GWTG-Stroke abstraction. Residents were provided with brief education on TOAST classification at the start of their stroke rotations. Assigned diagnoses were indicated using a drop-down menu of TOAST classifications as a required field in a discharge navigator form.

For reference standard ischemic stroke subtype diagnoses, two vascular neurology faculty members with expertise in TOAST classification (KTM, JLS) independently reviewed each patient’s medical record and imaging findings and assigned a TOAST stroke subtype classification. The experts were provided with the full formal classification system from the original TOAST publication and consulted this document when reviewing each case. Any difference in initial expert classifications were resolved by consensus discussion and re-review of records.

To characterize the patient population, the following characteristics were abstracted: age, sex, race, ethnicity, National Institutes of Health Stroke Scale (NIHSS) score, and pertinent medical history. To characterize the extent of the diagnostic work-up undertaken in the patients, the following data were abstracted from the chart: brain imaging and type [computed tomography (CT)/magnetic resonance imaging (MRI)], neck vessel imaging and type [computed tomography angiography (CTA)/magnetic resonance angiography (MRA)/ultrasound/digital subtraction angiography (DSA)], brain vessel imaging and type (CTA/MRA/ultrasound/DSA), cardiac echocardiogram and type [transthoracic echocardiogram(TTE)/transesophageal echocardiogram (TEE)], in-hospital cardiac rhythm monitoring, and transcranial doppler (TCD) with or without 30 min spontaneous embolus detection and bubble contrast studies.

### Statistics

The patient cohort was characterized using descriptive statistics, including mean and standard deviation for continuous variables and proportions for binary variables. The performance of clinical ischemic stroke etiology diagnosis compared with the reference standard was assessed by calculating sensitivity, specificity, and overall accuracy, both at the patient level and at the level of individual etiologies (some patients had more than one etiology). The accuracy of ischemic stroke etiology diagnoses in the EMR compared with the reference standard expert assessments was quantified by calculating the rate of matched diagnoses among all patients. (A kappa value was not calculated as kappa calculations presume that neither of the diagnostic sets is a reference standard.) Agreement of expert classifications prior to consensus discussion was assessed with Cohen’s kappa. Values between 0.00–0.20 were considered to indicate none to slight, 0.21–0.40 fair, 0.41–0.60 moderate, 0.61–0.80 substantial, and 0.81–1.00 as almost perfect agreement. The study sample size was calculated based on the following projected rates for key parameters in the comparison of EMR diagnosis to reference standard expert diagnosis: sensitivity 0.85, specificity 0.85, and prevalence of 25% for each major etiologic subtype. Within this framework, a sample size of 49 would be sufficient to narrow the 95% confidence interval around estimands of sensitivity and specificity to 0.2 (3, 4). The frequency and distribution of EMR diagnosis match and mismatch with the reference standard diagnosis was graphically evaluated with bubble plots.

## Results

The demographic and clinical characteristics of the 49 consecutive ischemic stroke patients meeting study entry criteria are shown in Table 1. Age was mean 72.3 ( ± 12.1), 53% were female, and presenting stroke deficit severity using the (NIHSS) score was median 3 (IQR 1–11). Race-ethnicities were: White, non-Hispanic 51%, Hispanic, white 16%, Black 16%, Asian 12%, and Other 4%. The three most common vascular risk factors were hypertension in 76%, dyslipidemia in 41%, and atrial fibrillation in 31%.

**Table 1:** Patient characteristics.

Patient characteristics	
Age, mean ± SD (*n* = 49)	72.3 ± 12.1
NIHSS, median (IQR) (*n* = 46)	3 (1–11)
**Sex (*n* = 49)**	
Male, *n* (%)	23 (47%)
Female, *n* (%)	26 (53%)
**Race (*n* = 49)**	
White, non-Hispanic, *n* (%)	25 (51%)
Hispanic, White	8 (16%)
Black, *n* (%)	8 (16%)
Asian, *n* (%)	6 (12%)
Other, *n* (%)	2 (4%)
**Medical history (*n* = 49)**	
HTN, *n* (%)	37 (76%)
Dyslipidemia, *n* (%)	20 (41%)
DM, *n* (%)	10 (20%)
CAD/MI, *n* (%)	7 (14%)
Atrial fibrillation, *n* (%)	15 (31%)
Current/recent smoker, *n* (%)	3 (6%)
Alcohol use, *n* (%)	0 (0%)
**Final reference standard TOAST diagnoses (patient-level) (*n* = 49)**	
LAA, *n* (%)	3 (6%)
SVD, *n* (%)	7 (14%)
CE, *n* (%)	19 (39%)
OTH, *n* (%)	5 (10%)
UND-M, *n* (%)	10 (20%)
UND-C, *n* (%)	5 (10%)
**Final reference standard TOAST diagnoses (etiology-level) (*n* = 60)**	
LAA, *n* (%)	9 (15%)
SVD, *n* (%)	11 (18.3%)
CE, *n* (%)	29 (48.3%)
OTH, *n* (%)	6 (10%)
UND-Comp, *n* (%)	5 (8.3%)

The elements of the diagnostic work-ups performed in the patient cohort are shown in Table 2. Brain parenchymal imaging was performed in all patients and included both MRI and CT in 55%, MRI only in 43%, and CT only in 2%. Imaging of cerebral vessels was performed in 98% of patients, as was imaging of cervical vessels. The most common vessel imaging modality was MRA, performed for cervical vessels and cerebral vessels in 82%, followed by CTA, performed in 51% of cervical and cerebral vessels. DSA was performed in 18% of patients. TCD was performed in 57% of patients, and included both monitoring for spontaneous microemboli and bubble study for right-to-left shunt in 51%. Echocardiography was performed in 92% of patients, including TTE alone in 86% and both TTE and TEE in 6%. Cardiac rhythm assessment included 12 lead electrocardiogram and continuous inpatient telemetry for ≥24 h in all patients. Hypercoagulable state testing was performed in 6 patients, including 2 (100%) of the patients whose EMR diagnosis was UND-C, 2 (50%) of the patients whose EMR diagnosis was Other defined cause, and 1 (25%) of the patients whose EMR diagnosis was not UND-C but whose expert diagnosis was UND-C.

**Table 2:** Work-up.

Studies	Total (*n* = 49)
**Brain imaging**	
NCCT only, *n* (%)	1 (2%)
MRI only, *n* (%)	21 (43%)
Both MRI and CT, *n* (%)	27 (55%)
**Neck vessel imaging**	
Any modality, *n* (%)	48 (98%)
CTA, *n* (%)	25 (51%)
MRA, *n* (%)	40 (82%)
DSA, *n* (%)	9 (18%)
Carotid duplex ultrasound, *n* (%)	3 (6%)
**Brain vessel imaging**	
Any modality, *n* (%)	48 (98%)
CTA, *n* (%)	25 (51%)
MRA, *n* (%)	40 (82%)
DSA, *n* (%)	9 (18%)
TCD brain ultrasound, *n* (%)	28 (57%)
**Cardiac echo**	
Any modality, *n* (%)	45 (92%)
TTE only, *n* (%)	42 (86%)
TEE only, *n* (%)	0 (0%)
TTE + TEE, *n* (%)	3 (6%)
**Cardiac rhythm assessment**	
EKG, 12 lead, *n* (%)	49 (100%)
Inpatient telemetry for ≥24 h, *n* (%)	49 (100%)
**Additional TCD studies**	
TCD 30 min embolus detection, *n* (%)	25 (51%)
TCD bubble study, *n* (%)	25 (51%)

The rates of reference TOAST diagnoses are shown at the bottom of Table 1. At the patient level, the most common final reference standard TOAST diagnoses were cardioembolic in 39%, undetermined due to more than one cause present in 20%, and small vessel disease in 14%. At the individual etiology-level the most common final reference standard TOAST diagnoses were cardioembolic in 48.3%, small vessel disease in 18.3%, and large artery atherosclerosis in 15.0%. The agreement of expert classifications prior to consensus discussion was moderate (kappa = 0.41).

**Figure 1:**
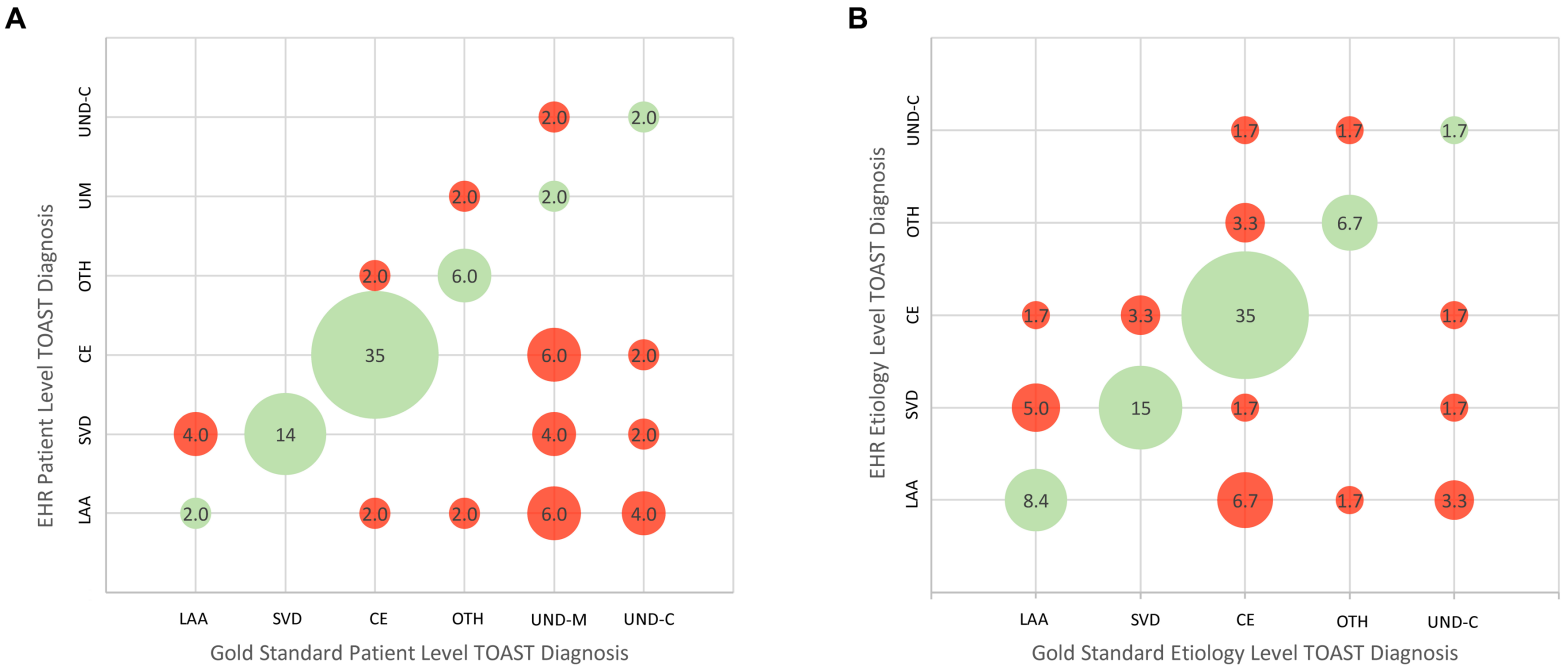
Bubble plots showing concordance and discordance between EMR/GWTG-Stroke etiologic diagnoses and expert reference diagnoses. **(A)** Patient-level analysis. **(B)** Etiology-level analysis. LAA, large-vessel atherothromboembolic; CE, cardioembolic; SVD, small vessel disease; OTH, other determined etiology; UND-M, undetermined–more than one cause undetermined due to presence of multiple potential etiologies; UND-C, undetermined despite complete work-up. The cohort had no cases of undetermined due to incomplete work-up.

Figure 1A shows the patient-level TOAST classifications documented in the EMR by the stroke neurology team compared with the reference standard diagnosis rendered by expert consensus. Among the 49 patients, 30 had diagnoses in the medical record matching the reference standard and 19 did not, yielding an accuracy rate of 61%. The bubble plot indicates the frequency of each correct and incorrect diagnostic pair. Among each of the six subtype diagnoses, specificity was generally high (84.8%–97.7%), but sensitivity suboptimal for LAA (33%), OTH (60%), UND-M (10%), and UND-C (20%). Positive predictive value was suboptimal for 5 of the 6 subtypes: LAA (12.5%), SVD (58.3%), OTH (75%), UND-M (50%), and UND-C (50%), though fair for CE (81%).

For the etiology-level analysis, the TOAST patient-level category of Undetermined (UND) was decomposed to its constituent parts. In the expert assessment, among the 15 patients with a TOAST patient-level diagnosis of Undetermined, the UND categorization reason was “more than one cause present” (UND-M) in 10 patients and “no identified cause present despite full work-up” (UND-C) in five patients. All 10 patients with more than one cause had two potential etiologies. When these were re-allocated into their individual categories, there were a total of 60 etiologic diagnoses. Figure 1B presents the ischemic stroke subtype classification agreement at the etiology level. Table 3 shows the numeric values for specificity, sensitivity, positive predictive value, and negative predictive value for each TOAST classification subtype. Among the 290 response options (1 correct and 4 incorrect for each of the 58 reference standard etiologies), chart diagnoses showed: sensitivity 66.7% (95CI 34.9%–90.1%); specificity 91.2% (95CI 80.0%–97.7%); positive predictive value 58% (95CI 41.9%–84.7%); and negative predictive value 90.8% (95CI 83.1%–96.1%). Considering etiologic judgments in binary manner, the medical record matched the reference standard in 40 and did not match in 20, yielding an accuracy rate of 67%. Among each of the five subtype diagnoses, specificity was generally high (86.3%–96.4%). Sensitivity was suboptimal for LAA (55.6%), OTH (66.7%), CE (72.4%), and UND-C (20%). Positive predictive value was acceptable for CE (84%) and suboptimal for the remainder: LAA (41.7%), SVD (64.3%), OTH (66.7%), and UND-C (33.3%).

**Table 3:** Medical record etiologic diagnoses compared with reference standard.

	All diagnoses	LAA	SVD	CE	OTH	UND-C
Sensitivity	66.7% (34.9%–90.1%)	55.6% (21.2%–86.3%)	81.8% (48.2%–97.7%)	72.4% (52.8%–87.3%)	66.7% (22.3%–95.7%)	20% (0.5%–71.6%)
Specificity	91.2% (80.0%–97.7%)	86.3% (73.7%–94.3%)	89.8% (77.7%–96.6%)	87.1% (70.2%–96.4%)	96.3% (87.3%–99.6%)	96.4% (87.5%–99.6%)
Positive predictive value	58% (41.9%–84.7%)	41.7% (22.5%–63.8%)	64.3% (42.8–81.2%)	84% (67.2%–93.1%)	66.7% (31.4%–89.7%)	33.3% (5.2%–82.1%)
Negative predictive value	90.8% (83.1%–96.1%)	91.7% (84.0%–95.8%)	95.7% (86.2%–98.7%)	77.1% (64.8%–86.1%)	96.3% (89.3%–98.8%)	93% (89.5%–95.4%)

**Figure 2:**
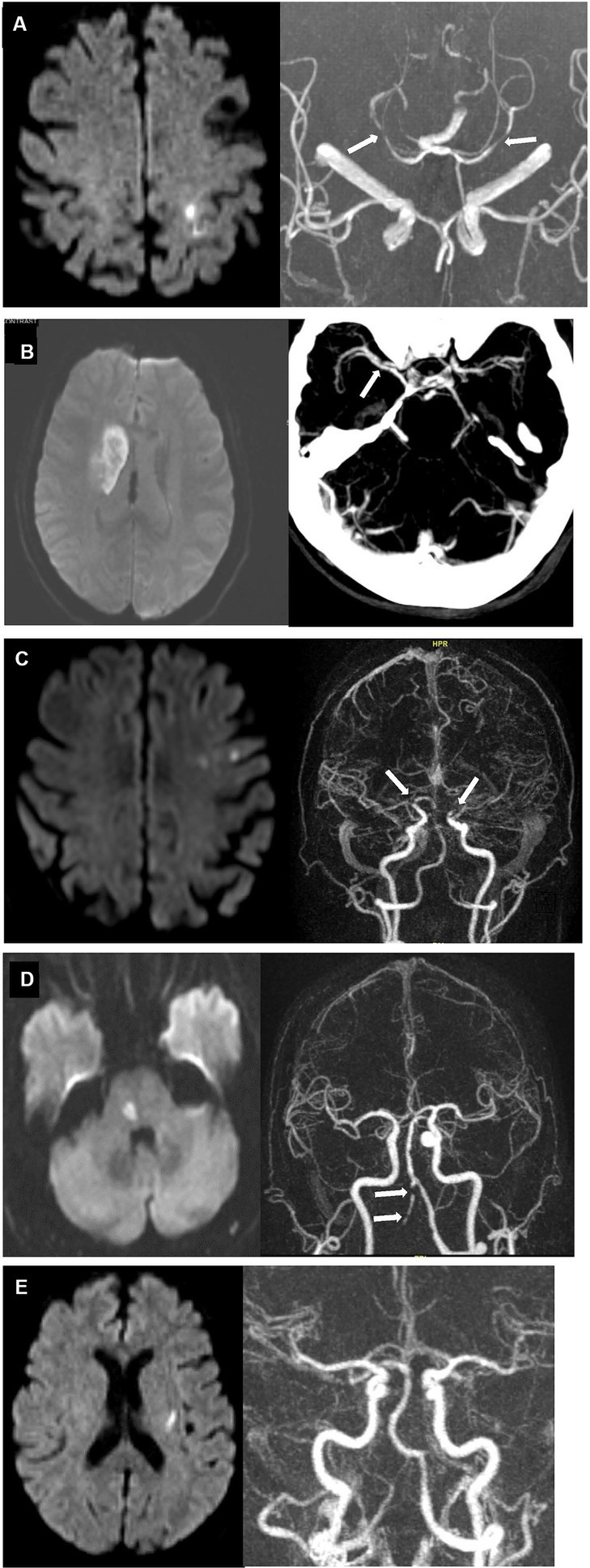
Case examples of discrepant EMR/GWTG-Stroke and reference standard TOAST diagnoses. **(A)** 80 years old man with history of hypertension, hyperlipidemia, and possible aortic stenosis presented with right-sided weakness and numbness. Diffusion MRI (left) showed new infarct involving left paracentral and superior parietal lobules. MRA (right) showed focal moderate stenoses in the right P2 posterior cerebral artery and mild stenosis in the left posterior cerebral artery (white arrows) but not stenoses in the anterior circulation supplying the infarcted territory. TTE showed only possible mild aortic stenosis. Inpatient cardiac telemetry monitoring for 72 h showed no rhythm abnormality. TOAST etiologic diagnoses entered in EMR/GWTG-Stroke were both LAA and CE. Reference standard diagnosis was cryptogenic despite complete work-up (embolic stroke of uncertain source; UND-C). **(B)** 68 years old woman with history of hypertension, hyperlipidemia, and diabetes presented with left facial droop, slurred speech, and mild left arm weakness. DWI MRI (Left) showed 4.2 cm long (anteroposterior) right striatal infarct. Severe focal narrowing of the right middle cerebral artery M1 segment was seen on CTA (right, white arrow). TTE and extended inpatient cardiac telemetry monitoring were unremarkable. TOAST etiologic diagnosis entered in EMR/GWTG-Stroke was SVD. But reference standard diagnosis was LAA. **(C)** 74 years old woman presented with word finding difficulties for 1 week. DWI MRI (left) showed multiple, small subacute infarcts in left frontal lobe and centrum semiovale. MRA (right) showed high-grade, near-occlusion of both the distal left and distal right supraclinoid internal carotid arteries (white arrows). TTE showed mitral annulus calcification. Extended inpatient cardiac telemetry monitoring was unremarkable. TOAST etiologic diagnosis entered in EMR/GWTG-Stroke was LAA, but reference standard diagnosis was cryptogenic—more than one source present (LAA and CE) (UND-M). **(D)** 71 years old man with history of hypertension and hyperlipidemia presented with left hand numbness. DWI MRI (left) showed acute right paramedian pontine infarct. MRA (right) showed severe multifocal atherosclerotic stenoses of right vertebral artery (white arrows). TTE showed mild diastolic dysfunction. Extended inpatient cardiac telemetry monitoring was unremarkable. TOAST etiologic diagnosis entered in EMR/GWTG-Stroke was SVD. Reference standard diagnosis was cryptogenic—more than one source present (SVD and LAA) (UND-M). **(E)**. 73 years old woman with history of hypertension and hyperlipidemia presented with right hand numbness. Diffusion MRI (left) showed small acute left basal ganglia infarct. MRA (right) and TTE were unremarkable. Inpatient cardiac telemetry captured atrial fibrillation. TOAST etiologic diagnosis entered in EMR/GWTG-Stroke was CE. But reference standard diagnosis was cryptogenic—more than one source present (SVD and CE) (UND-M).

An error pattern analysis of cases with discrepant EMR/GWTG-Stroke and reference standard TOAST diagnoses is shown in Table 4 and illustrative cases are shown in Figure 2.

**Table 4:** Error patterns in assigning TOAST etiologic diagnosis in electronic medical record.

Gold standard TOAST classification (*n*)	Correct EMR TOAST classifications	Incorrect EMR TOAST classifications	Error type	Error description
LAA (3)	1	2 (SVD –2)	Considering macune to be lacune (1)	Considered large, deep, striatocapsular infarct in patient with intracranial atherosclerotic disease to be of small vessel origin.
			Not considering subtle and follow-up imaging findings (1)	Initial imaging showed not only clear cerebral peduncle infarct but also subtle occipital infarct; initial vessel imaging suboptimal but follow-up showed LAA in vertebral artery.
CE (19)	17	2 (LAA-1; OTH – 1)	Considering acute occlusion as LAA rather than embolus and not considering mitral annular calcification as CE source (1)	Considered acute occlusion as LAA rather than embolus and not considering mitral annular calcification as CE source.
			Not considering PFO a CE source and not recognizing acute phase reactant cause of factor VIII elevation (1)	Did not a large shunt PFO as a CE source and did not recognize acute phase reaction as cause of factor VIII elevation.
OTH (5)	3	2 (LAA-1; UND-M-1)	Considering procedure-related embolization as LAA (1)	In patient with embolic stroke during diagnostic angiography, classified as LAA due to presumed aortic arch atherosclerosis without any direct evidence that arch atherosclerosis was present or severe.
			Assigning CE mechanism based on deficit pattern and topography, not CE source (1)	In hypercoagulable state patient with quadrantanopia and anterior choroidal artery infarct, also indicated CE mechanism based on clinical syndrome and infarct location despite no CE source identified.
UND-C (6)	2	4 (LAA-2; CE-1; SVD-1)	Considering ESUS as cardioembolic (1)	Classified patient with a single cortical infarct or a prior infarct in another vascular territory as cardioembolic despite the absence of an identified cardiac source.
			Considering low-risk sources as medium-high risk sources (3)	In patients without TOAST-level evidence for an etiology, physicians had tendency to identify a stroke mechanism using weaker evidence rather than assign correct cryptogenic rating.
UND-M (8)	0	8 (LAA-3; CE-3; SVD-1; UND-C-1)	When LAA present, ignoring competing CE sources (heart block, mitral annular calcification) (3)	In patients with LAA, ignoring competing CE sources (heart block, mitral annular calcification).
			Considering lacunar infarcts in AF patient as CE (3) or SVD (1) but not both	In patients with known atrial fibrillation and a single penetrator stroke, classifying as only have a possible CE SVD mechanism or rather than both CE and SVD possible mechanisms.
			Focusing upon non-stroke CNS disease (1)	In patient presenting with small cerebellar infarct in setting of active CNS lymphoma, not attending to potential causes of infarct including AF and hypercoagulable state.

## Discussion

This study found that ischemic stroke subtype diagnoses based on the TOAST classification system and entered into the electronic medical record and the GWTG-Stroke were often inaccurate when compared against reference diagnoses rendered by experts in TOAST application. Four of every 10 patients were found to have not been accurately classified. Clinician-rendered diagnoses generally showed good specificity, but suboptimal sensitivity, especially for large artery atherothromboembolism, undetermined due to multiple etiologies present, and undetermined despite complete work-up. Positive predictive values were suboptimal for all ischemic stroke subtypes.

Several distinct types of errors by clinicians in rendering TOAST etiologic diagnoses were observed. Several reflected clinician dispositions to attribute causality based on weak rather than firm, algorithm-endorsed evidence, including ascribing causality to low risk sources (e.g., mild atherostenosis in a large artery); and attributing embolic topography infarcts to cardioembolism despite no cardiac source being identified. Others reflected clinician unfamiliarity with somewhat nuanced diagnostic distinctions, such as considering large, deep infarcts to be due to single-penetrator, small vessel disease (lacunes), rather than large artery, multi-penetrator disease (macunes).

Another source of error were aspects of the TOAST algorithm that are now known to be sub-optimal. For example, the TOAST algorithm classifies mitral valve prolapse and mitral annulus calcification as medium-risk sources of cardioembolism, but subsequent studies have shown they are at most low risk sources (5–9); and the TOAST algorithm classifies patent foramen ovale as a medium-risk source of cardioembolism, but subsequent studies have shown that patent foramen ovale with a large shunt and/or an accompanying atrial septal aneurysm is a high-risk source of cardioembolism (10).

We are not aware of prior studies investigating the accuracy of TOAST-based ischemic stroke etiology diagnoses in the Get with the Guidelines – Stroke registry. However, our results align with a study from the Third China National Stroke Registry which found an even lower accuracy rate for site-rendered etiologic diagnoses compared with the reference standard of centralized expert adjudicators (11). Our results are also consonant with numerous studies that have found imperfect agreement among raters using the TOAST instrument (12, 13). Several studies have shown that inter-rater agreement is improved when the TOAST algorithm is updated to incorporate diagnostic modalities that have emerged since the instrument’s development 30 years ago (8, 14, 15). However, no revised version of the TOAST algorithm has received wide adoption. Several alternative ischemic stroke etiologic classification systems have been developed since TOAST was first promulgated and to varying degrees incorporate more updated pathophysiologic understanding and diagnostic test types. These include: the Causative Classification of Stroke; the ASCOD Phenotyping system; the Chinese ischemic stroke classification; and the Subtypes of Ischaemic Stroke Classification System (SPARKLE) (16). These tools may have the potential to improve accuracy of ischemic stroke subtype diagnosis. But all contain complicated algorithms designed for use by expert researchers, making it impractical to adopt them into routine practice unmodified. We were not able to find studies comparing clinical practice diagnoses with expert rater diagnoses for these instruments. In addition to these causative classification systems designed to identify the mechanism of ischemic stroke, simpler phenotypic classification systems designed to identify whether a large, medium, or small vessel is occluded in ischemic stroke have been developed (17–19). While useful, including for guiding acute reperfusion therapy decision-making, these systems do not provide etiologic information necessary for long-term secondary stroke prevention management.

This study has limitations. First, it is a single-center study. Although the patient population was diverse in race-ethnicity and stroke mechanism, replication in a larger set of centers is desirable. Second, sample size was moderate, limiting precision of performance rate estimates, again making replication in a larger sample desirable. Formal size calculation indicated acceptable power for analysis of overall agreement but suboptimal power for analysis of individual stroke mechanisms. Third, the clinicians documenting TOAST diagnoses in the electronic medical record did not undergo detailed annual retraining and certification in use of TOAST. However, the lack of formal training reflects usual practice nationally and we are unaware of an accepted formal TOAST classification certification process. Fourth, the expert raters in the current study were not blinded to the initial EMR diagnoses. However, they were instructed to not let these affect their own assignments in any manner. Fifth, consensus diagnosis by expert raters is not an absolute ground truth. However, it is the methodologically most rigorous approach to TOAST diagnosis assignment. For this reason, expert consensus diagnoses were used as the reference standard in the current study as in prior studies comparing less rigorous with more rigorous TOAST assessments (11, 20, 21). Sixth, the rates and types of inaccuracies in community settings may differ from the current study in an academic stroke center.

In conclusion, clinical practice ischemic subtype diagnoses using TOAST and entered into the GWTG-Stroke registry were not fully accurate in 4 of every 10 patients. This suboptimal performance in part reflects clinician unfamiliarity with the formal TOAST classification algorithms and in part reflects that some elements of the TOAST system have been rendered outmoded by diagnostic advances in the last three decades. As a result, clinicians are using a non-operationalized, intuitive approach to mechanism classification, which requires no special training, but can be a source of poor inter-rater reliability and poor correspondence with actual pathophysiology. This inaccuracy, if similarly present at other centers, would hamper the ability of the GWTG-Stroke national registry to provide dependable insights into subtype-related aspects of care. These findings suggest a need to develop improvements to ischemic stroke etiology classification implementable in routine clinical practice. Efforts are under way to develop artificial intelligence systems that assist in the application of current etiologic classification systems (22, 23). However, while worthwhile, this approach will not address the issue that aspects of the current classification systems have been rendered outmoded by advances in diagnostic technologies and pathophysiologic understanding. An attractive approach would be to develop an updated etiologic classification system that is sufficiently pragmatic and efficient to implement in routine clinical practice, incorporates the more advanced diagnostic tools and knowledge now available, and has algorithmic logic that can be directly embedded in electronic medical records. Ideally such a system could be endorsed and then continuously updated and curated by a consensus group of experts in neurovascular nosology.

## Data availability statement

The original contributions presented in the study are included in the article/supplementary material, further inquiries can be directed to the corresponding author.

## Ethics statement

The studies involving humans were approved by the University of California, Los Angeles, Los Angeles (UCLA) Institutional Review Board. The studies were conducted in accordance with the local legislation and institutional requirements. Written informed consent from the patients/participants or patients/participants' legal guardian/next of kin was not required to participate in this study in accordance with the national legislation and the institutional requirements.

## Author contributions

CR: Formal analysis, Investigation, Methodology¸ Writing – original draft, Writing – review & editing. KM: Formal analysis, Investigation, Writing – review & editing. LS: Writing – review & editing. JS: Conceptualization, Data curation, Formal analysis, Funding acquisition¸ Investigation, Methodology, Project administration, Resources, Supervision, Validation, Visualization, Writing – original draft¸ Writing – review & editing.
